# Association-Based Analysis of Verticillium Wilt Resistance in a Bi-Parental Hop (*Humulus lupulus* L.) Population for Marker Development in Breeding

**DOI:** 10.3390/plants15111667

**Published:** 2026-05-29

**Authors:** Lucija Luskar, Martin Waldinger, Nicholi J. Pitra, Alexander Feiner, Sebastjan Radišek, Jernej Jakše, Andreja Čerenak

**Affiliations:** 1 Department for Plants, Soil and the Environment, Slovenian Institute of Hop Research and Brewing, Cesta Žalskega Tabora 2, 3310 Žalec, Slovenia; 2 Department for Agronomy, Biotechnical Faculty, University of Ljubljana, Jamnikarjeva 101, 1000 Ljubljana, Slovenia; 3 Hopsteiner, Simon H. Steiner, Hopfen, GmbH, Auhofstrasse 18, 84048 Mainburg, Germany; 4 Hopsteiner, S.S. Steiner, Inc., 725 5th Avenue, New York, NY 10022, USA; 5 Plant Protection Diagnostics Laboratory, Slovenian Institute of Hop Research and Brewing, Cesta Žalskega Tabora 2, 3310 Žalec, Slovenia

**Keywords:** hop, GWAS, Verticillium wilt resistance, SNP marker, marker-assisted selection

## Abstract

Verticillium wilt of hop (*Humulus lupulus* L.), caused by the soil-borne pathogen *Verticillium nonalfalfae*, is a devastating disease with no effective chemical control. In European hop-growing regions, breeding resistant cultivars is the most effective strategy. The lack of response differences in earlier studies suggests constitutive resistance. We therefore conducted a genome-wide association study (GWAS) using a phased hop genome assembly to improve detection of Verticillium resistance loci. A bi-parental population of 142 genotypes, derived from a cross between resistant Wye Target and susceptible BL2/1, was phenotyped for Verticillium wilt resistance and genotyped by sequencing. Association analyses with five statistical models (MLM in TASSEL 5, MLM, MLMM, FarmCPU and BLINK in GAPIT) did not identify any significant SNPs; however, several candidate loci were identified using exploratory threshold, particularly in the phase 2 genome assembly, including a wall-associated kinase (WAK) consistently detected across both genome phases and all models. GWAS results were further assessed with a Random Forest model, which identified SNPs of high feature importance and showed adequate predictive power (accuracy ≈ 0.4, correlation ≈ 0.8) for preliminary breeding screening. These findings provide an initial set of candidate markers and exploratory prediction models for Verticillium wilt resistance in hop, representing a valuable genomic resource for future marker-assisted selection and breeding strategies.

## 1. Introduction

In the face of rapid climate change, breeding for resilience should be a top priority for all crops. The breeding of new cultivars to replace older ones must take into account not only yield and chemical compounds, but also robust resistance to both biotic and abiotic stress [1,2]. Hop (*Humulus lupulus*) breeding primarily focuses on developing cultivars for the brewing industry, with an emphasis on yield, resin content and essential oil quantity and composition, as well as increased resistance to major pathogens. The process is complex due to the species’ dioecious nature, perennial growth habit, long juvenile phase, and obligate outcrossing, which result in high levels of genetic heterozygosity. At the genomic level, the challenge is further intensified by a large, repetitive genome (2.5 to 3.4 Gb) [3,4,5] and centromere-driven chromosomal instability. As recently highlighted by Horáková et al. [6], centromeric repeat diversity leads to frequent meiotic errors and chromosome-specific instability, which, alongside widespread non-Mendelian segregation, can disrupt standard inheritance patterns [7].

Verticillium wilting of hop is a disease, manifested in the interaction between soil-borne fungus *Verticillium nonalfalfae* or *Verticillium dahliae* and susceptible hop cultivars [8]. The lethal type of disease is caused by *V. nonalfalfae*, highly virulent pathotypes or strains that trigger severe symptoms like chlorosis, necrosis, wilting and plant tissue death, consequently causing the death of the plant in the same or the next season [9,10,11]. The *V. nonalfalfae* strain with increased virulence in hop was first discovered in the United Kingdom (UK) in 1933 [12] followed by outbreaks in Slovenia in 1997 [13], Germany in 2005 [14], Czech Republic in 2017 [15] and most recently in Belgium [16]. According to recent reports, Verticillium wilt (VW) affects about 40% of hop growing area in Germany [17], the country with the greatest hop acreage [18]. Given that no chemical prevention is available against soil-borne diseases, breeding and planting resistant or tolerant cultivars is the only defensive strategy.

A Ve1-like receptor, homologous to the well-characterized tomato Ve1 [19], has been reported in hop [20] and was experimentally validated to recognize the Verticillium effector Ave1 [21]. In contrast, homology searches within the *V. nonalfalfae* genome [22] failed to identify the Ave1 effector sequence, suggesting that the mechanism of recognition and resistance in hop is likely different [23]. Response studies of resistant and susceptible cultivars revealed faster and stronger defence mechanisms in susceptible cultivars, including tylose formation, accumulation of lignin and suberin on xylem walls, mechanisms that lead to development of symptoms [23,24,25,26]. In the infected roots of the susceptible cultivar, elevated levels of defence-related proteins—including chitinase, β-glucanase, thaumatin-like protein, peroxidase, and germin-like protein—were found [26]. The findings imply that susceptibility to Verticillium infection is caused by a failure in the effectiveness of the responses rather than the lack of immune responses [26]. Although the immune response is more vigorous in the susceptible hop cultivar, certain defence mechanisms are more active in the resistant cultivar [25,26]. However, it is important to note that none of these genes or transcripts have proved to be reliable enough to be used as potential resistance breeding markers.

Hop is a highly heterozygous, clonally propagated crop characterized by extended linkage disequilibrium, in which disease resistance is typically selected at the clonal or family level rather than through repeated multi-parent recombination. The use of bi-parental populations remains one of the most powerful and practical approaches for genetic discovery in hop [27,28,29,30]. By focusing on the progeny of two contrasting parents, researchers can maximize the phenotypic variance for specific traits, such as disease resistance, within a controlled genetic background. Historically, genome research in hops was primarily driven by the need to identify molecular markers for marker-assisted breeding. Early efforts focused heavily on linkage mapping; however, these maps were often limited by low marker density and poor resolution [30,31,32].

To map hop resistance to *V. nonalfalfae*, Jakše et al. [32] utilized a Wye Target × BL2/1 full-sib F_1_ population to construct a linkage map of 203 markers across ten linkage groups. This study successfully identified VW resistance QTL on LG03, explaining 24.2–26.0% of the phenotypic variance, yet the limited marker density and reliance on older marker types resulted in large intervals that hindered practical marker-assisted selection. While bi-parental QTL mapping remains appropriate for locus detection, genome-wide association studies (GWAS) offer a complementary, exploratory framework to capture marker effects across breeding-relevant haplotypes [27]. This approach is particularly relevant in hop, where the prerequisites for stable recombination-based mapping are frequently violated. Zhang et al. [7] identified widespread non-Mendelian segregation and structural heterozygosity that can induce pseudo-linkage and complicate marker ordering. Furthermore, Horáková et al. [6] linked centromeric repeat diversity to aberrant chromosome behaviour and instability, suggesting that recombination fractions in hop F_1_ progenies may not reliably reflect physical linkage [33].

Henning et al. [27] established a successful precedent for hop research by utilizing a bi-parental population and GBS to perform a GWAS for downy mildew resistance. This methodology has become an increasingly vital tool in the hop industry as it combines the high-resolution advantages of association mapping with the statistical reliability of structured families. By using bi-parental populations, researchers can effectively mitigate the false discovery rates typically associated with traditional GWAS in unstructured natural populations. While the recent development of hop reference genomes has greatly enhanced our mapping capabilities [3,4], the GWAS approach remains a highly efficient strategy for identifying a vast number of segregating SNPs and narrowing down candidate genomic regions.

In the present study, we revisited the bi-parental cross between the resistant cultivar Wye Target (WT) and the susceptible genotype BL2/1. While previous research on this population identified a QTL [32], it failed to pinpoint the underlying major loci or provide markers closely associated enough for practical breeding. To address this, we employed a GWAS framework within this segregating family to identify marker–trait associations directly. This approach serves as a necessary alternative to traditional linkage mapping, which often loses critical genetic information in hop due to prevalent segregation distortion and atypical meiosis. By leveraging the novel Apollo hop genome assembly and accounting for relatedness, we integrated both GWAS and genomic prediction (GP) to reveal specific genomic sites associated with *V. nonalfalfae* response. Our goal is to provide actionable genetic information that, once validated across diverse genotypes, will facilitate the development of more effective genomic selection models for hop breeding programmes.

## 2. Results

### 2.1. Phenotyping

**Figure 1:**
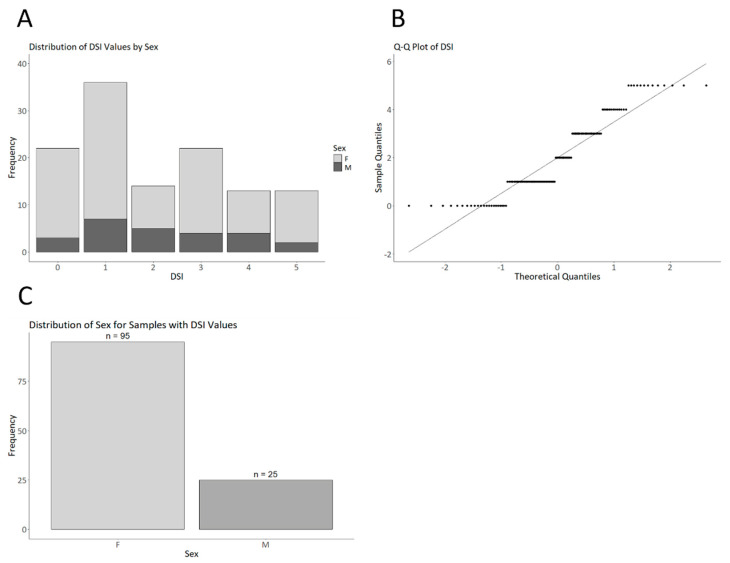
(**A**)—Frequency distribution of disease severity scores (DSI) for 120 genotypes from cross Wye Target × BL2/1 including sex distribution for each DSI score. (**B**)—Q-Q plot of DSI with diagonal line presenting expected distribution. (**C**)—Sex frequency of the included genotypes.

Comprehensive phenotyping was performed to track the inheritance of VW resistance in the F_1_ mapping population. The study included a total of 142 F_1_ full-sib genotypes derived from a cross between the resistant English cultivar Wye Target and the susceptible breeding line BL 2/1. This population consisted of 78 offspring previously phenotyped by Jakše et al. [32] and 66 genotypes newly propagated for this study. From the latter group, we successfully obtained new disease severity index (DSI) data for 42 offspring. For the GWAS analysis, a total of 120 genotypes with confirmed DSI scores were included, comprising the F_1_ offspring and parental lines. The most often assigned score was 1 (36), indicating 1–20% leaf surface with symptoms, followed by 22 genotypes with score 0 and 3, 14 with score 2 and 13 with score 4 and 5 (Figure 1A). The Q-Q plot of the DSI indicates a deviation from normality (Shapiro–Wilk test: W = 0.89242, *p*-value = 8.191 × 10^−8^), with notable deviations from the reference line at both ends, suggesting a positive skewness of the DSI (Figure 1B). The sex ratio of 120 assessed genotypes was 3.8:1 (female:male) (Figure 1C) (Supplementary File S1).

### 2.2. Sequencing and Mapping

On average, offspring were sequenced at the amount of 0.4 Gb of raw sequencing data, while parents were sequenced at the depth of 21.7 Gb. Sequencing reads were mapped to both phases of the Apollo genome. The average percentage of mapped reads from the offspring for both phases was 99.2% with an average number of 3 million reads per sample. Parents were sequenced using whole-genome sequencing (WGS) approach, resulting in much higher coverage (approximately 30×) and yielding about 98 million and 96 million reads for p1 and p2 respectively (Supplementary File S2).

### 2.3. Filtering of VCF and SNP Distribution

The generated variant call files (VCF) were imported to TASSEL 5 and filtered for a maximum proportion of missing data per site, MAF and minimum and maximum heterozygosity (Supplementary File S3, Table S1). The VCF of the studied family that were used for statistical models contained 27,277 and 36,127 SNP positions for p1 and p2, respectively.

Phase 2 genome assembly had a higher number of SNP filtered positions compared to phase 1, while the number of non-filtered sites was greater in phase 1 than in phase 2 (Supplementary File S3, Table S1). For phase 1 (filtered), the highest density is on chromosomes 7 and 2, respectively, whereas phase 2 has the highest density on chromosomes 4, 3 and 6 (based on average SNP distance, Supplementary File S3, Figure S1). The average read depth (DP) for SNPs was greater than 500 in both phases (Supplementary File S3, Table S2).

### 2.4. Principal Component Analysis

**Figure 2:**
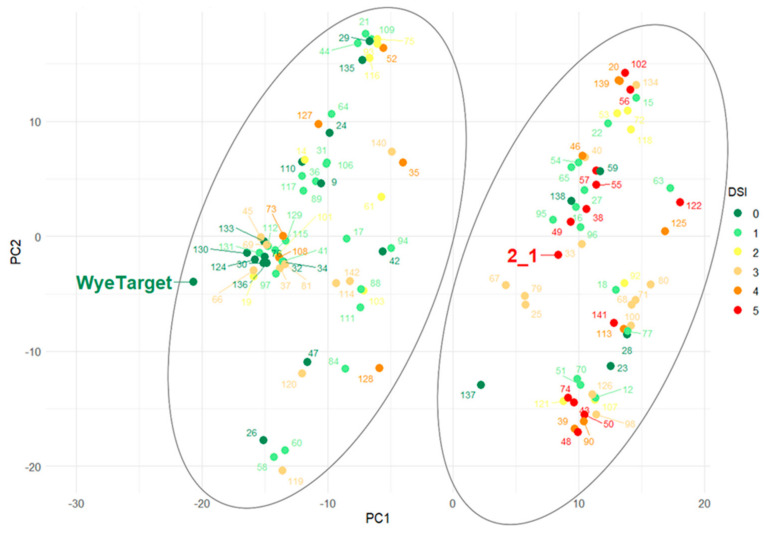
PCA plot of 120 genotypes from the WT × BL2/1 family on phase 1. The colour indicates DSI score and k-means clustering (k = 2) of the family indicated by two ellipses. Mother Wye Target is in cluster 1 and father BL 2/1 in cluster 2.

The results of principal component analysis (PCA) showed that the first principal component (PC1) of p1 had an eigenvalue of 147.01, explaining 5.8% of the total genetic variance, while the first six principal components together accounted for roughly 17% of the total variance, indicating a moderate level of population structure within the dataset. PCA for p2 showed that PC1 had an eigenvalue of 423.82, accounting for 11.6% of the total variance. The first six principal components together accounted for about 29.2% of the total variance. In both phases, plot of PC1 vs. PC2 formed two groups, each containing a parent of the family (Figure 2). The susceptible father 2/1 group included all genotypes with the highest susceptibility (DSI 5) to VNA, whereas other DSI levels were evenly distributed between both groups. Grouping into two clusters by two PC was detected also by K-means clustering (k = 2) for both phases (Figure 2, p2 in Supplementary File S4).

### 2.5. GWAS Models

**Figure 3:**
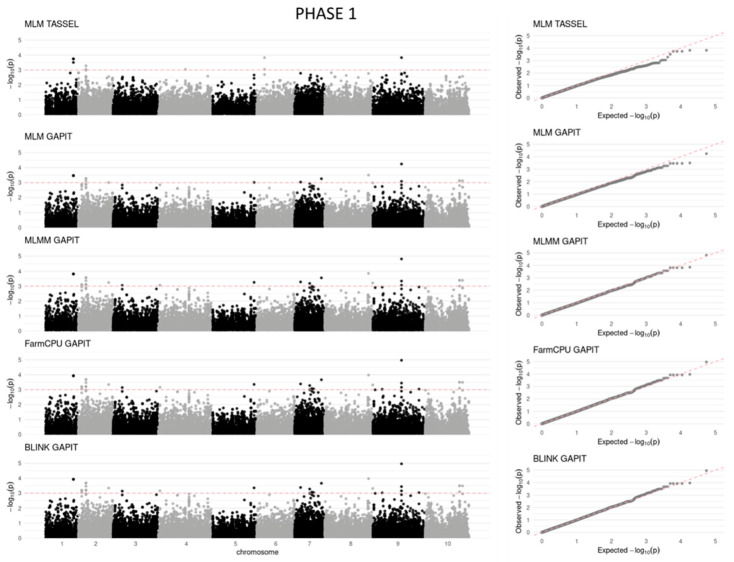
Genome-wide association analysis of WT × BL2/1 family, mapped to phase 1, for DSI in interaction with *Verticillium nonalfalfae*. Manhattan plot of each model and corresponding Q-Q plot. Dashed line on Manhattan plot at −log_10_p = 3 represents exploratory threshold.

**Figure 4:**
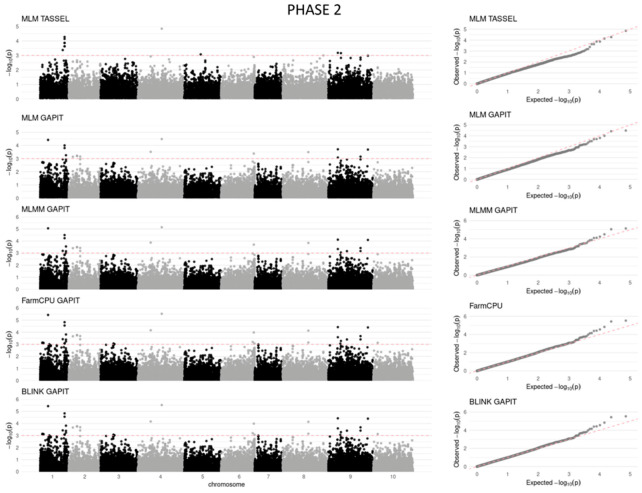
Genome-wide association analysis of WT × BL2/1 family, mapped to phase 2, for DSI in interaction with *Verticillium nonalfalfae*. Manhattan plot of each model and corresponding Q-Q plot. Dashed line on Manhattan plot at −log_10_p = 3 represents exploratory threshold.

GWAS were performed using MLM in TASSEL 5, and using MLM, MLMM, FarmCPU and BLINK in GAPIT. The Q-Q plots show that the observed *p*-values follow the expected uniform distribution, with a slight deviation at the tail (Figure 3 and Figure 4). Bonferroni and fdr corrections did not identify any genome-wide significant associations, likely reflecting the limited sample size and genetic structure of the bi-parental population. To balance stringency and detection power, an exploratory significance threshold (*p* < 0.001) was applied. Combined with cross-model consistency, this approach consistently highlighted a set of candidate associations that warrant further validation—36 SNPs in p1 and 43 SNPs in p2 (Figure 3 and Figure 4; Supplementary File S5 for p1, Supplementary File S6 for p2).

**Figure 5:**
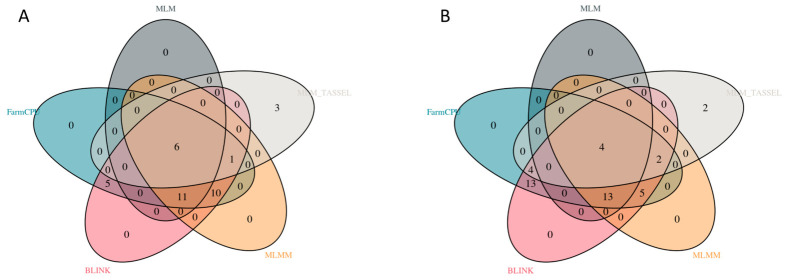
Venn-diagram showing the intersections of SNPs (*p* < 0.001) between the models. (**A**)—phase 1, (**B**)—phase 2.

Six SNPs were identified as candidate positions across all models (S01_170688839, S01_170688849, S01_170688858, S01_170688863, S02_47001453, S09_177368328) for p1 (Figure 5A) and four for p2 (S01_162046452, S01_162046458, S04_159980635, S09_64850003, Figure 5B). Remarkably, BLINK and FarmCPU detected the same set of SNPs, with total overlap between the two methods (Figure 5A,B). These models also identified the largest number of candidate SNPs, with comparatively stronger association signals. The MLM implemented in TASSEL uniquely identified three SNPs on p1 (S06_48630581, S06_48630585, S04_167151172) (Figure 3 and Figure 5A) and two for p2 (S05_110100766, S08_270007223) that were not detected by any of the GAPIT models (Figure 4 and Figure 5B). The GAPIT models showed a high degree of consistency in SNP detection, with differences observed mainly in the magnitude of statistical significance attributed to the overlapping set of SNPs. *p*-values of SNPs S04_159980635 (0.0000030; −log_10_p = 5.524) and S01_52552992 (0.0000037; −log_10_p = 5.430) of p2 approached the Bonferroni threshold (0.0000014; −log_10_p = 5.860) in FarmCPU and BLINK models (Figure 4). Genomic positions that are common to both phases were identified at the distal end of chromosome 1 and in the central region of chromosome (chr) 4 (Figure 3 and Figure 5).

### 2.6. Identification of Consensus SNPs Across GWAS Models

To prioritize the most consistently supported candidate associations, we focused on SNPs that were detected across all five GWAS models under the exploratory threshold (Figure 5). For reporting summary statistics (Table 1), we present results from the BLINK and FarmCPU because these models produced the lowest *p*-values and showed identical statistical outputs.

The multi-model GWAS approach, utilizing the intersection of five statistical frameworks, highlighted a set of candidate SNP-trait associations with VW response (DSI) (Table 1). A notable observation of this study was the identification of a putative QTL-cluster on chr1, consistently detected across both p1 and p2 datasets using exploratory thresholds. In p1, this locus is defined by a cluster of markers (S01_170688839-01_170688863) associated with a 3-dehydroquinate dehydratase and a cluster of Wall-associated receptor kinases (WAKs). Similarly, in the p2 phase, exploratory associations were identified in the same genomic vicinity (S01_162046452 and S01_162046458), also targeting WAKs and serine/threonine protein kinases (IREH1). The consistent detection of this region across both phases and all five GWAS models designates the chr1 kinase cluster as a highly plausible candidate region for VW resistance, warranting prioritization for future functional validation.

In addition to the candidate resistance-associated loci, the p1 analysis highlighted a putative susceptibility-associated locus on chr2 (S02_47001453). This marker showed a relatively strong positive effect (β = 0.92), indicating that the alternative allele at this position may be associated with increased disease severity. Furthermore, a candidate association showing a comparatively large effect was detected on chr9 (S09_177368328) in the p1 phase. This region corresponds to a genomic region annotated with receptor-like protein kinase 7, which showed the highest negative effect size (β = −0.897). The consistent detection of these regions across all five GWAS models highlights these kinase-rich clusters as plausible annotation-based candidates for further investigation into VW resistance. Overall, these loci represent candidate regions supported by cross-model consistency; however, validation in independent material and functional work will be required to determine causality and assess utility for marker-assisted selection.

### 2.7. Random Forest Prediction

**Figure 6:**
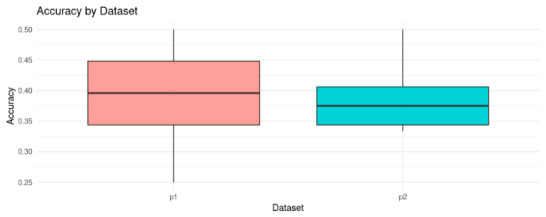
Accuracy for samples tested in Random Forest.

**Figure 7:**
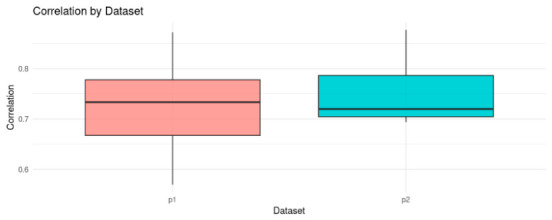
Correlation for samples tested in Random Forest.

The Random Forest model with 80% of data as training and 20% as testing set achieved a mean prediction accuracy of 0.396 for p1 and 0.383 for p2, with p2 showing less variation in accuracy across the 10 different data splits (Figure 6). The mean correlation between observed and predicted values was 0.730 for p1 and 0.750 for p2 (Figure 7).

**Figure 8:**
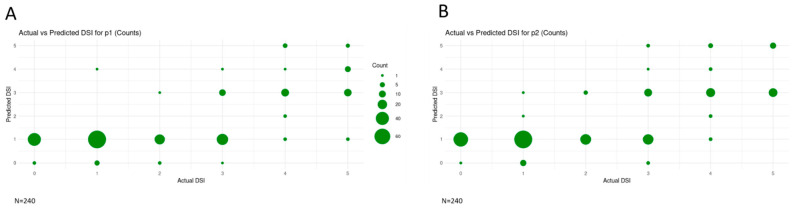
Plotted observed Disease Severity Index (DSI) scores per tested genotype vs. predicted DSI with predictions were made by Random Forest model. (**A**)—phase 1, (**B**)—phase 2.

Tested samples were evaluated for their prediction and compared to the actual DSI (Supplementary Files S7 and S8). Among 240 (24 genotypes in one data split × 10 data splits) predictions, the model predicted resistant genotypes with high accuracy, while predictions for susceptible genotypes showed greater variability (Figure 8). The model trained on p2 data performed better in predicting susceptibility than the p1 model. None of the genotypes classified as highly susceptible genotypes (DSI 4 and 5) were predicted to be resistant (DSI 0), and none of the resistant genotypes (DSI 0 and 1) were predicted to be highly susceptible (DSI 5). In p2, resistant genotypes 22 and 51 were incorrectly predicted as DSI 2 and 3, respectively, whereas in p1, one genotype (sample 64) was mispredicted as DSI 4. Notably, a sample that was mispredicted in p1 (64) was accurately classified in p2, and those misclassified in p2 (22 and 51) were correctly predicted in p1.

### 2.8. Consensus Markers Between Random Forest Importance and GWAS Results

**Figure 9:**
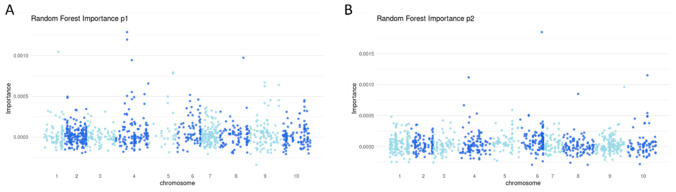
Importance plots for Random Forest prediction model. (**A**)—phase 1 and (**B**)—phase 2.

**Figure 10:**
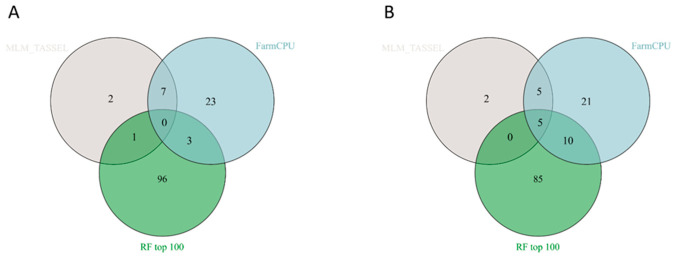
Venn-diagrams showing SNPs intersections between GWAS models and Random Forest (RF) prediction model. (**A**)—phase1 and (**B**)—phase 2.

We examined the top 100 SNP positions ranked by variable importance from the Random Forest model (Figure 9, Supplementary File S9 for p1, Supplementary File S10 for p2) and compared their overlap [34] with SNPs (*p* < 0.001) found using the GWAS models (Figure 10). The intersection of these SNP sets was then used to identify common loci detected by multiple methods. SNPs with a significance of *p* < 0.001, as well as the top 10 SNPs identified by RF, were selected and mapped on the annotated genome with 46 SNPs for p1 and 50 for p2 (Supplementary File S11). 24 SNPs in p1 and 35 in p2 resulted in annotated genomic regions (46 for p1 and 57 for p2) within the ±50 kbp window. The extracted annotated sequences were analyzed using Blast2GO (Supplementary File S12) and visualized in Supplementary File S13.

The variable importance analysis from the RF model highlighted key regions on chr4 for p1 and p2; these regions were also found to be highlighted in p2 for the GWAS models (Figure 9). The SNP S04_167151172 (linked to serine/threonine protein kinase) was identified by both the MLM in TASSEL and among the top 100 SNPs in the RF model (Supplementary File S11). Furthermore, several other SNPs on this chromosome of p1 (S04_118125400, S04_118125402, S04_164615890, S04_32673844) were in the top 10 of the RF model. Notably, three of the top 100 SNPs identified by RF (S07_117498606, S09_285506050, S10_212010288) also overlapped with those selected by the FarmCPU model with exploratory threshold of *p* < 0.001 (Figure 10). While the RF model identified S01_136627768 on chr1 as important, the GWAS models highlighted an upstream region at S01_170688xxx. Furthermore, the RF model indicated additional regions of importance on chr5 and 8 (Figure 9, Supplementary File S11) that were not detected by GWAS models.

The highlighted regions of RF for the p2 are on chr4, 6, 8, 9 and 10 (Figure 9). The consensus SNPs across models, GWAS and RF, are S01_149973437, S01_162046452, S01_162046469, S04_159980635, S09_6485003. Their possible linkage to some annotated genes are listed in Supplementary File S12 and graphically presented in Supplementary File S13.

### 2.9. Overlap with Previous Studies

Microsatellite markers linked to a previously identified Verticillium wilt resistance QTL (HlAGA6, HlAGA8 and EMHL052 [32]) were localized on chromosome 6 of the Apollo genome (Supplementary File S14). The best match for all three was found on chr6, spanning 10.902.851 bp and 9.784.311 bp in p1 and p2 respectively. This overlaps with SNP which was highlighted by MLM TASSEL 5 (S06_4863058x, p1); however, the screened area of ±50 kbp revealed no annotated genes. Despite the previously reported effect of this QTL, explaining approximately 24–26% of the phenotypic variance [32], it was not consistently detected across GWAS models in the present study.

Despite the previous discovery of HlVe1 homologues (similar to tomato Ve genes) as putative resistance gene analogues in hop [20], our analysis found no highlighted SNPs in this region (Table 2). Interestingly, this region is located approx. 37 Mbp upstream of a previously described QTL by Jakse et al. (2013) [32] on chr6 (Supplementary File S13).

## 3. Discussion

### 3.1. Challenges of Phenotyping

One of the main challenges in hop breeding is the difficulty of evaluating a vast number of new and highly heterozygous offspring in field trials, which reduces selection efficiency. Obtaining reliable phenotypic assessments for resistance to VW in hop is a labour-intensive and time-consuming process that requires the expertise of experienced personnel. The development of methods for preselecting thousands of new plantlets before they reach the maturity for field planting could significantly facilitate the hop breeding. By identifying genetic markers associated with specific traits or diseases, GWAS enhances our understanding of host–pathogen interactions and improves disease-resistant breeding strategies [35].

### 3.2. Phased Reference Genome in GWAS

For the first time chromosome-level, phased Apollo genome assembly (PRJNA1082089) [4] was used as a reference to map the sequencing data. Compared to p2 (27,277), p1 (36,127) contained about one third more filtered SNPs. Due to more SNP in p2, the map was also denser for a particular phase (Figure 2). A similar number (27,174) of properly segregating SNPs were also used for hop GWAS in the study of Havill et al. [29] when working with Cascade hop genome [3]. When comparing the performance of GWAS models in our study, p2 yielded lower *p*-values and a greater number of highlighted SNP associations compared to p1.

### 3.3. Performance of GWAS Models

To address the challenges of classical linkage mapping in hop, where extreme heterozygosity and segregation distortion often destabilize recombination-based maps [6,27,30,31,33], we employed a GWAS-style mixed-model framework as a robust alternative [27]. By anchoring markers to physical coordinates on the Apollo assembly, we bypassed the need for a genetic map and performed direct marker-trait association testing while controlling for family structure. Given the family-based structure, and expected polygenic basis of VW resistance, the statistical power to detect associations with moderate effect site was limited. Therefore, we adopted an exploratory significance threshold (*p* < 0.001), which provides a compromise between stringency and sensitivity in similar exploratory GWAS [27,36,37]. Cross-model overlap was used as a prioritization criterion to reduce model-specific false positives, but not as a substitute for formal validation. BLINK and FarmCPU demonstrated the best statistical performance and produced identical results, most likely due to their similar iterative approaches that alternate between fixed and random effect models. Both methods are designed to minimize confounding effects caused by population structure and relatedness, which is particularly effective in populations with simple structure as in our case [38]. While sex-linked loci are known in hop, the potential confounding effect of sex was mitigated by the inclusion of kinship and PCA covariates in the GWAS models, which account for the population structure associated with parental origin. No enrichment of significant associations was detected in known sex-linked genomic regions. BLINK and FarmCPU also detected all SNPs identified by GAPIT MLM and MLMM (Figure 5A,B). TASSEL 5 MLM identified additional SNPs, that were not highlighted by these models, including a SNP linked to previously discovered VW resistance QTL [32]. However, the value of these associations would require further investigation.

### 3.4. Comparison of GWAS to Genomic Prediction

While acknowledging the inherent limitations of genomic prediction in bi-parental populations, we integrated machine learning analysis as a complementary method to GWAS for SNP prioritization based on feature importance. For p1, the RF model assigned importance to different SNP positions compared to the GWAS models, with no overlap in the top SNPs identified by each approach. In contrast, for p2, there were five SNP positions shared between the GWAS and the RF top 100. For p2, two SNPs consistently identified as important across all models were previously detected by five GWAS models and linked to a kinase cluster (Supplementary File S11). While there is some overlap between GWAS and machine learning approaches, particularly for p2, the most important SNPs can vary significantly depending on the method used [39].

### 3.5. Key Candidate Genes and Functional Insights from GWAS

In general, the candidate SNPs identified in this study are predominantly involved in plant defence, stress response, and signal transduction pathways, including a number of receptor-like kinases, disease resistance proteins, and transcriptional regulators. The region common to both phases and consistently detected across all GWAS models (chr1, Table 1, Supplementary Files S12 and S13) corresponds to a genomic region annotated with wall-associated kinases, a unique subfamily of receptor-like kinases that play fundamental roles in the perception of external stimuli and activate defence signalling pathways [40]. WAK proteins possess a typical cytoplasmic Ser/Thr kinase signature and provide communication between the extracellular matrix and the cytoplasm [41]. WAKs have been found to recognize damage-associated molecular patterns (DAMPs), which comprised the pectin and oligogalacturonide molecules that are released from the plant cell wall damaged by pathogen attack. Transgenic *Arabidopsis* plants overexpressing WAK1 are more resistant to *Botrytis cinere* [42]. In our study, the WAK-associated markers on chromosome 1 showed consistent negative effects across both phases (p1: β = −0.71; p2: β = −0.63), indicating that these alleles are associated with lower disease severity in this population. The modulation of WAK expression in hop may be influenced by miRNA-617, which has targets in WAK transcripts and had lower expression in WT after inoculation with *V. nonalfalfae* compared to susceptible cultivar. A similar parallel can be drawn with hlu-miR156e, significantly downregulated miRNA of WT with targets in squamosa promotor binding gene transcripts [43], which are putatively associated with genomic region on p2, chr1 by GWAS models (Supplementary File S13). Squamosa promoter binding proteins, in addition to functioning as transcriptional activators involved in leaf development, vegetative phase change, flower and fruit development, plant architecture, shoot maturation, and gibberellin signalling, have also been linked to responses to fungal toxins [44]. Interestingly, Padgitt-Cobb et al. [3] identified the most copies of defence genes in syntenic blocks on Cascade chr8, which corresponds to chr1 in the Apollo genome where WAK and squamosa genes were identified and may represent a biologically plausible candidate region related to VW response.

### 3.6. Genetic Architecture of Resistance

Resistance traits were introduced into the UK hop breeding programmes in the 1930s from two distinct sources of American wild hops (*H. lupulus* var. *neo-mexicanus*), named Y90 and AA 7 [45]. Following the outbreaks of lethal pathotype PG2 in Slovenia, research efforts for study interactions between hop and Verticillium were initiated. The majority of the research has focused on the Wye Target cultivar, which is related to AA 7 resistance source, either by comparing it to the susceptible cultivar Celeia or by examining populations derived from crosses between Wye Target and the susceptible line BL 2/1 [20,24,25,26,32,46,47]. Studies of compatible and incompatible hop–VNA pathosystems indicate that the resistance relies on constitutive rather than induced resistance mechanisms [24,26]. This might reflect in inheritance of dominant resistance alleles, possibly with polygenic contributions as reported in other studies [32,45].

Although a major QTL on chromosome 6 was previously identified in this population by Jakše et al. [32], explaining approximately 24–26% of the phenotypic variance, this region was not consistently detected across GWAS models in the present study. This discrepancy likely reflects differences between linkage mapping and association-based approaches. Linkage mapping relies on within-family co-segregation in a defined cross, whereas GWAS depends on LD and allele frequency in the analyzed sample, which can reduce power to recover the same peak. In addition, only a subset of the original mapping population was retained in this study (78), while additional genotypes were newly propagated, potentially altering allele frequencies and reducing the power to detect the same locus. Furthermore, the higher marker density and distinct statistical frameworks used in GWAS may lead to the detection of adjacent or alternative candidate regions rather than direct recovery of previously identified QTL peaks.

In cotton, resistance to *V. dahliae* is recognized as a complex trait controlled by many genes, each having a small effect which complicates breeding for broad-spectrum resistance using targeted genetic selection [48,49]. Our GWAS results indicate similar patterns that are also in hop family of Wye Target and BL2/1, as several loci across the genome were highlighted. Clustering in PCA indicates that highly susceptible genotypes (DSI 5) are genetically distinct and grouped with the susceptible parent (Figure 2), whereas the rest of the population shows more variation and overlap, potentially implying more complex or quantitative basis for resistance.

### 3.7. Genomic Prediction for Enhanced Breeding

Recent advances in GP have shown that integrating GWAS can significantly enhance predictive accuracy [39,50,51,52]. The ensemble structure of RF, which aggregates predictions from multiple decision trees built on bootstrap samples and random subsets of predictors, enhances both predictive accuracy and robustness, and is particularly effective at capturing nonadditive genetic effects [53]. In our study, we selected the top 1000 SNPs identified by FarmCPU/BLINK for use in a RF model as a practical subset for model construction, based on preliminary testing and computational considerations [51]. As the Random Forest model was trained on GWAS-prioritized SNPs, this integrated approach functions as a multi-step prioritization framework rather than an independent validation of association signals. The resulting prediction accuracy in our bi-parental study was approximately 0.4, which is comparable to previous studies on wheat resistance to leaf spotting diseases (average accuracy = 0.53; [54]). The prediction of strawberry resistance to *Verticillium dahliae* ranged from 0.38 to 0.53 depending on the year and regression method [55]. The highest reported prediction accuracy for hop was R1 resistance to powdery mildew in genomic selection using rrBLUP that reached about 0.89 [56], whereas alpha-acid content in hop was predicted with an accuracy of 0.69 using gBLUP [57]. Although predictive accuracy is crucial in practical breeding, a strong correlation (approx. 0.7) can be sufficient to eliminate highly susceptible genotypes early in the breeding pipeline. We acknowledge that predictive models developed in a single bi-parental cross cannot be generalized to diverse breeding populations due to limited recombination and strong linkage disequilibrium. Nevertheless, from a breeding standpoint, the prediction model developed for the family Wye Target × BL2/1 thus serves as proof-of-concept for within-family selection. For broader applicability, the model should be validated and refined on a more heterogeneous population. To achieve this, additional phenotyping efforts will be required to improve its predictive power and ensure robustness across diverse genetic backgrounds.

## 4. Materials and Methods

### 4.1. Phenotypic Data Collection

**Figure 11:**
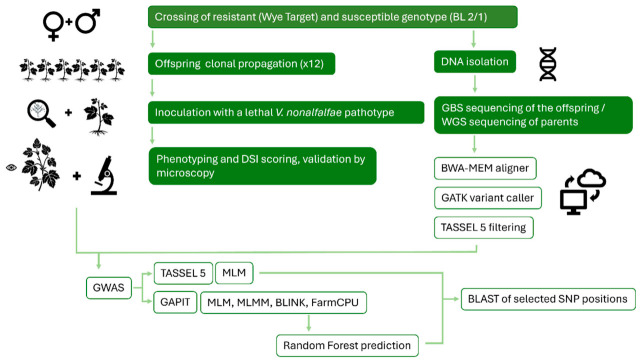
Workflow scheme.

The F_1_ mapping population derives from a cross between the VW highly resistant English cultivar Wye Target and the susceptible Slovene male breeding line 2/1 (Figure 11). This mapping family was originally established and phenotyped for VW resistance by Jakše et al. [32] and has been continuously maintained since then. Of the original F_1_ population, 78 genotypes have been successfully preserved and retained their corresponding disease severity index (DSI) scores from the original phenotyping. Additionally, 66 genotypes were newly propagated and prepared for a DSI evaluation experiment to supplement the existing data. The specific year of phenotyping for each genotype is provided in Supplementary File S1. Phenotyping was conducted using a standardized inoculation protocol, controlled environmental conditions, and a consistent DSI scoring system across all experiments. Although genotypes were evaluated in different years, the use of identical experimental procedures minimized potential batch effects; however, minor residual variation cannot be completely excluded. Hop plants (full sib-F_1_ genotypes, parents and reference cultivars: susceptible Celeia, susceptible Fuggle, moderately resistant Wye Challenger) were vegetatively propagated using softwood cuttings in a greenhouse. Each genotype, represented by 12 clones was inoculated with a mixture of four *V. nonalfalfae* isolates, all previously identified as lethal hop pathotype PV1, PG2 genotype [11], by ten-minute root-dipping in a fungal spore suspension (5 × 10^6^ conidia/mL), while 12 clones of each reference cultivars were also mock inoculated using sterile water [58]. Inoculated plants were cultivated in the glasshouse after being potted into a commercial potting substrate (S04-2004 Topf/Pikier, Gramoflor, Germany). Disease phenotyping was performed using the DSI, and infection was confirmed by pathogen re-isolation, according to an established protocol [32,58]. Plants were assessed weekly for foliar symptom severity after the appearance of the first symptoms using a 0–5 scale, where 0 indicates no visible leaf symptoms, 1 corresponds to 1–20% wilted leaf area, 2 to 21–40%, 3 to 41–60%, 4 to 61–80%, and 5 to 81–100% wilted leaf area. Plant infection was confirmed by re-isolation of the fungus from roots and stems. Cut pieces of the vascular tissue were placed on potato dextrose agar and the presence of mycelium development was examined by light microscopy after 3–5 days. Final genotype assessment was expressed as disease severity index (DSI) calculated as mean wilt score of the infected plants on last foliage assessment. The DSI normal distribution was tested using the Shapiro–Wilk normality test [59]. Sex of the plants was determined by visual observations in a field trial [32] and additionally confirmed by sex marker (hPb-365890) in qPCR [60].

### 4.2. Sequencing, Alignment, and Variant Calling

Approximately 50 mg of leaf material from each genotype was sampled with a 5 mm punch, dried in 96 well plate and sent to LGC Genomics (Berlin, Germany) for DNA extraction and sequencing. The library preparation with ApeKI for the offspring was performed according to LGS’s protocols. The parent’s library (Wye Target and breeding line 2/1) was generated by DNA fragmentation by enzyme mix Allegro Targeted Genotyping kit (TECAN genomics, Redwood City, CA, USA) and sequenced by Illumina NextSeq2000 using XLEAP-SBS chemistry (Illumina, San Diego, CA, USA), 250 paired-end reads while the offspring library was sequenced by Illumina NovaSeq 6000 (Illumina, San Diego, CA, USA), 150 paired-end reads. Data were delivered in fastq format, quality and adapter trimmed (deposited in the National Center for Biotechnology Information (NCBI) Sequence Read Archive (SRA) under BioProject ID PRJNA1357051).

BWA-MEM [61] was used to align sequencing reads to haplotype-resolved, chromosome-level assembly of the hop cultivar Apollo [4]. This assembly represents the diploid genome as two distinct, phased sequences—referred to as phase 1 (p1) and phase 2 (p2)—each corresponding to one of the two homologous haplotypes These phases do not constitute different genome versions but rather the two distinct parental sequences of the same chromosome-level assembly. Samtools [62] ‘sort’ and ‘flagstats’ were used to sort bam files and to generate a mapping report. Picard [63] was used to perform ‘AddOrReplaceReadGroups’ and ‘MarkDuplicates’ in mapping files. Variant calling was performed using the Genome Analysis Toolkit (GATK) ‘HaplotypeCaller’, ‘CombineGVCFs’, and ‘GenotypeGVCFs’ workflows [64,65], in accordance with the best practices as recommended.

Genotyped variant call format (VCF) files were imported into the TASSEL 5 software [66] for downstream analysis. Variant filtering parameters for the VCF file were based on previously published studies on hop resistance [29,67], and subsequently refined to account for the specific characteristics of our study population. Briefly, variants were filtered based on the following criteria: a maximum proportion of missing data per site of 35.5%, a minor allele frequency (MAF) threshold of 0.05, a minimum heterozygosity of 0.2, and a maximum heterozygosity of 0.7. SNP density was visualized by ‘CMplot’ [68]. Principal component analysis (PCA) and kinship analysis were performed in TASSEL 5. PCA and k-means (‘kmeans’, 2 clusters) analysis were visualized with ‘ggplot2’ [69] in RStudio (2024.12.1).

### 4.3. Multi-Model GWAS

The filtered VCF file was fed into five genome-wide association study (GWAS) algorithms to ensure comprehensive and reliable identification of marker-trait associations. For the mixed linear model (MLM) implemented in TASSEL 5, the filtered VCF file was integrated with kinship matrix and PCA data. The numerically imputed genotype file and PCA data for the models executed in GAPIT were exported from TASSEL 5 and subsequently imported into RStudio to perform MLM [70], multi-locus mixed model (MLMM [71]), Bayesian-information and linkage-disequilibrium iteratively nested keyway (BLINK [38], and Fixed and random model Circulating Probability Unification (FarmCPU [72]) analyses. All generated datasets were imported to RStudio for downstream analysis and visualization using the ‘ggplot2’ [69] package. The quantile–quantile (Q-Q) plots generated from the observed against expected −log10p -values were used to evaluate the performance of the statistical models. The Bonferroni-corrected significance thresholds were calculated at −log10p = 5.74 (α/nr. of SNPs; 0.05/27,277 = 1.83 × 10^−6^) for p1 and −log10p = 5.86 (α/nr. of SNPs; 0.05/36,127 = 1.38 × 10^−6^) for p2. To account for multiple testing, *p*-values were also adjusted using ‘p.adjust, method = “fdr”’ function in RStudio. Exploratory threshold of *p* < 0.001 was used to select SNPs exceeding this level in at least one of the five GWAS models, allowing for cross-model comparison and candidate locus identification [36,37]. To compare the overlap of SNPs identified across different models, the RStudio package ‘VennDiagram’ [73] was used to visualize intersections.

### 4.4. Genomic Prediction

Random Forest (RF) training procedure was selected as the prediction model due to its nonparametric nature and its ability to capture complex, non-linear relationships between genetic markers and phenotypes [74]. It was performed by an algorithm implemented in the R-package ‘ranger’ [75], with the top 1000 SNPs from FarmCPU/BLINK models and DSI specified as response variable. The number of SNPs was selected as a pragmatic subset for model construction, based on preliminary testing, balancing model complexity and computational efficiency, rather than as a formally optimized parameter. Samples were split into a 0.8 training and 0.2 testing ratio, with reproducibility ensured by specifying a random seed through the set.seed() function. The model was constructed with 2000 trees and within 10 independent data splits to calculate the permutation-based variable importance. Model performance was assessed by calculating prediction accuracy, correlation between observed and predicted values, and mean variable importance. Accuracy was calculated from a confusion matrix by dividing the sum of correctly classified samples by the total number of samples. Pearson correlation coefficient was computed between actual and predicted DSI values to assess the linear relationship between observed and predicted outcomes.

### 4.5. BLAST Analysis of Candidate Genes

Candidate SNPs were selected using the GWAS models’ *p*-value (*p* < 0.001) in conjunction with the top 10 candidate SNPs from genomic prediction. Selected SNPs were saved as .bed file, imported to CLC Genomics Workbench Version 25.0 (Qiagen, Aarhus, Denmark) and annotated to the genome for each phase separately. The flanking regions ± 50 kbp around SNPs were extracted by tool Extract annotated regions and the list of annotated genes in region was then subject for BLAST (Blast2GO, 1.5.1) analysis. Functional annotation of genomic regions surrounding highlighted SNPs was performed using Blast2GO Command Line Interface (CLI) version 1.5.1 [76]. The .seqtable data was adopted for downstream analysis.

Microsatellite markers that are linked to previously identified QTL linked to VW resistance and Humulus Ve-like genes were downloaded from NCBI to CLC Genomics Workbench Version 25.0. Sequences were BLASTed against the Apollo genome, p1 and p2, using programme blastn to find their position. Hits with longest HSP length and lowest E-value were used for locating and scanning for nearest SNPs.

## 5. Conclusions

To our knowledge, this is the first GWAS conducted using a chromosome-level, haplotype resolved hop genome assembly. The genome is represented by two phased assemblies (phase 1 and phase 2), each corresponding to one haplotype of the same diploid individual. By performing independent read mapping and GWAS analyses against each haplotype assembly, we were able to directly assess how haplotype resolution influences the detection of genotype–phenotype associations. The study employed five GWAS models and one prediction model to associate SNPs found in the family of WT and BL2/1 with the response to VW of hop. Potential candidate genes and prediction models were used to address the urgent need for genetic assessment of resistance/susceptibility to the *V. nonalfalfae*.

The genes linked to the highlighted SNPs are predominantly involved in plant defence, stress response, and signal transduction pathways, including several receptor-like kinases, disease resistance proteins, and transcriptional regulators (Supplementary File S12). Given the prevalence of putative small-effect loci, prediction models may offer a more promising tool for selection breeding, since they can capture complex, non-linear associations. Although this is a bi-parental study and the findings require further validation across broader genetic backgrounds, it presents significant progress in the hop GWAS with results laying the groundwork for further studies not only on VW resistance, but also on other trait associations. These findings expend our current knowledge about the potential mechanisms underpinning hop resistance to VW and provide new genes and loci for future pathogen tolerance improvement strategies. By providing an initial set of candidate SNPs and genes, this study highlights the potential of GWAS and GP for future breeding applications in this complex crop.

## Figures and Tables

**Table 1:** Consensus exploratory SNPs (*p* < 0.001) across GWAS models, their genomic position, gene annotations in the ±50 kbp region and associated phenotypic effects on Disease Severity Index (DSI).

SNP	Phase	Blast2GO Annotation	Apollo Gene ID	*p*-Value ^1^	MAF	Effect (β)	Res/Sus Allele
S01_170688839	P1	3-dehydroquinate dehydratase type I; wall-associated receptor kinase 2-like; wall-associated receptor kinase; wall-associated receptor kinase 2-like;	HUMLU.APOL.r2.01P1G0285900	1.18 × 10^−4^	0.49	−0.71	C/G
S01_170688849			HUMLU.APOL.r2.01P1G0286000				C/T
S01_170688858			HUMLU.APOL.r2.01P1G0286100				A/G
S01_170688863			HUMLU.APOL.r2.01P1G0286200				T/A
S02_47001453	P1	/	/	2.10 × 10^−4^	0.25	0.92	T/C
S09_177368328	P1	protein ALP1-like;	HUMLU.APOL.r2.09P1G2612400	1.00 × 10^−4^	0.354	−0.897	A/G
		receptor-like protein kinase 7;	HUMLU.APOL.r2.09P1G2612500				
		retrovirus-related Pol polyprotein from transposon RE2;	HUMLU.APOL.r2.09P1G2612600				
		chloroplastic group IIA intron splicing facilitator CRS1;	HUMLU.APOL.r2.09P1G2612700				
		retrovirus-related Pol polyprotein from transposon RE1 isoform X1;	HUMLU.APOL.r2.09P1G2612800				
S01_162046452	P2	probable serine/threonine protein kinase IREH1;	HUMLU.APOL.r2.01P2G0283600	1.00 × 10^−4^	0.47	−0.63	C/G
S01_162046458		Wall-associated receptor kinase	HUMLU.APOL.r2.01P2G0283700	1.58 × 10^−4^	0.49	−0.65	A/T
S04_159980635	P2	/	/	3.29 × 10^−5^	0.29	−0.82	C/T
S09_64850003	P2	ruBisCO large subunit-binding protein subunit beta, chloroplastic-like	HUMLU.APOL.r2.09P2G2520600	1.97 × 10^−4^	0.44	0.72	G/A

^1^ values and effect sizes (β) are reported based on the BLINK and FarmCPU models.

**Table 2:** List of predicted HLVe1 genes and its positions in the Apollo phased genome.

Annotation on Apollo Genome	Gene	Accession	Phase	Chromosome	Position
HUMLU.APOL.r2.06P1G1622900	HlVe1-2A	KJ647426	P1	Chr6	3,101,491–3,103,256
HUMLU.APOL.r2.06P1G1622800	HlVe1-2B	KJ647427	P1	Chr6	3,099,685–3,101,429
HUMLU.APOL.r2.07P1G1924800	HlVe1-1	KJ647424	P1	Chr7	12,265,591–12,269,993
HUMLU.APOL.r2.07P2G1944000	HlVe1-2A HlVe1-2B	KJ647426 KJ647427	P2	Chr7	21,022,992–21,025,999
HUMLU.APOL.r2.07P2G1929700	HlVe1-1	KJ647424	P2	Chr7	12,056,941–12,060,272

## Data Availability

All relevant data are summarized in the manuscript’s tables and figures, and included as supplementary files. Raw sequence data is deposited in the National Center for Biotechnology Information (NCBI) Sequence Read Archive (SRA) under BioProject ID PRJNA1357051.

## Supplementary Materials

The following supporting information can be downloaded at: https://www.mdpi.com/article/10.3390/plants15111667/s1, Supplementary File S1: Text file containing phenotypes used in models. Columns contain data about taxa, year of phenotyping, disease severity index (DSI) and sex. Supplementary File S2: Text file containing sequencing data by sample and mapping report to both phases of Apollo genome. The file contains sample name, total nucleotides, total sequences and for each phase number of mapped reads, percent of mapped reads, number of properly paired reads, percent of properly paired reads, number of singletons and percent of singletons. Supplementary File S3: Filtering of VCF files and SNP distribution data. Supplementary File S4: Two figures of PCA analysis of Apollo genome phase 2 filtered variant file. Supplementary File S5: SNP names, positions (CHR and BP) and *p* values by different model for phase 1. Each sheet represents selection of SNPS with *p* < 0.001 by particular model (in bold) and its *p*-values of other models for comparison. Supplementary File S6: SNP names, positions (CHR and BP) and *p* values by different model for phase 2. Each sheet represents selection of SNPS with *p* < 0.001 by particular model (in bold) and its *p*-values of other models for comparison. Supplementary File S7: Data of Random Forest prediction for samples in 10 different testing sets for phase 1. Data includes sample, their actual DSI and their predicted DSI in particular data split (Split). Supplementary File S8: Data of Random Forest prediction for samples in 10 different testing sets for phase 2. Data includes sample, their actual DSI and their predicted DSI in particular data split (Split). Supplementary File S9: Variable importance from the Random Forest model of phase 1 with SNP (Variable) and its mean importance (Importance). Supplementary File S10: Variable importance from the Random Forest model of phase 2 with SNP (Variable) and its mean importance (Importance). Supplementary File S11: File containing two sheets, first is the list of selected SNP from p1 and second for p2, with indication if the SNP was selected by FarmCPU or MLM Tassel or Random Forest variable importance top 10 SNPs and indication if it was among top 100 SNPs. Supplementary File S12: File containing two list of genes annotated on Apollo genome (Query) (phase 1 on sheet p1 and phase 2 on sheet p2) that are in the window +- 50 kbp from our SNP positions and their BLAST output Seq. Description and SNP (SNP_name) that are related to the Query. Supplementary File S12 consists also of the following columns, derived from blas2go search: Seq. Length (sequence length of the protein), #Hits (number of database matches), min. eValue (lowest expectation value indicating match significance), mean Similarity (average sequence similarity across hits), #GOs (number of associated Gene Ontology terms), GOs (the specific Gene Ontology terms), Enzyme Codes (enzyme classification numbers), and InterProScan (domain and motif annotations identified by InterProScan). Supplementary File S13: Two figures of Manhattan plots of SNPs with *p* < 0.001 from all GWAS models and top 10 important SNPs from Random Forest. Supplementary File S14: List of markers associated with Verticillium wilt resistance QTLs and their positions in the Apollo phased genome.

## Abbreviations

The following abbreviations are used in this manuscript:

DSI	disease severity index
GWAS	genome-wide association study
PCA	principal component analysis
QTL	quantitative trait locus
RF	Random Forest
SNP	single-nucleotide polymorphism
VCF	variant call format
VNA	*Verticillium nonalfalfae*
VW	Verticillium wilt
WAK	wall-associated kinase
