# Supplementary material for: Association-Based Analysis of Verticillium Wilt Resistance in a Bi-Parental Hop (Humulus lupulus L.) Population for Marker Development in Breeding
Source: Plants (Basel). 2026 May 29;15(11):1667. doi: 10.3390/plants15111667 (PMC13259542; doi:10.3390/plants15111667)
Supplement: Supplementary file 1 [file plants-15-01667-s001.zip › Supplementary_file_S13.pdf]

Supplementary file S13: Two figures of Manhattan plots of SNPs with p<0.001 from all GWAS models and top 10 important SNPs from Random Forest.

P1

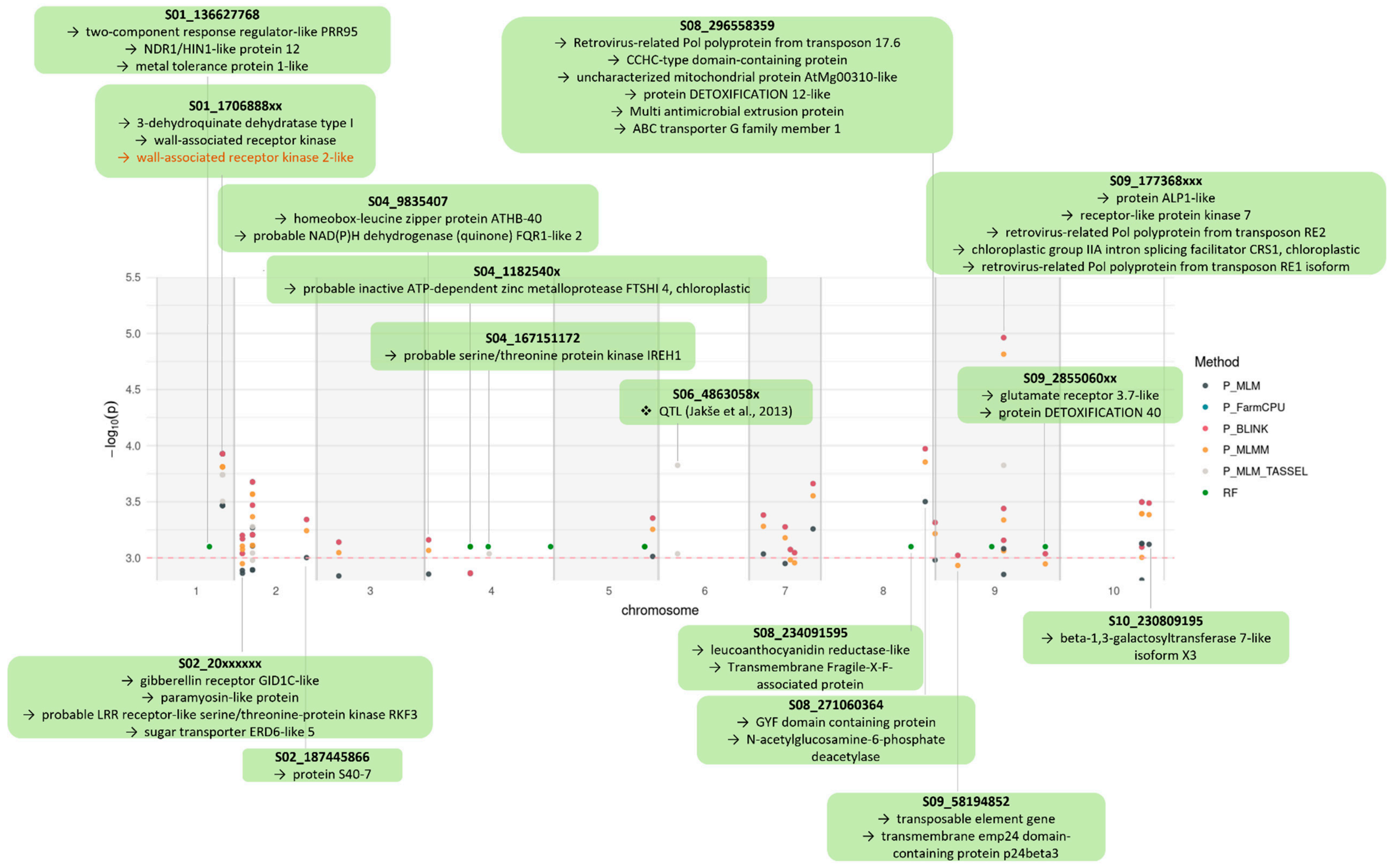

Figure S13.1: Manhattan plot of phase 1 with all the GWAS models with SNPs with p<0.001 (above dashed pink line) and top 10 important SNPs from Random Forest with artificial p value (-$\log_{10}$p=3.1*) for illustration. FarmCPU and BLINK p-values are denoted as BLINK values since they overlay. SNPs with annotated genes in the ±50 kbp region are marked and its BLAST descriptions from Supplement_11 are noted (→). ❖ indicates authors annotation. Orange colour in the gene name indicates that the gene has SNPs on its coding region.*

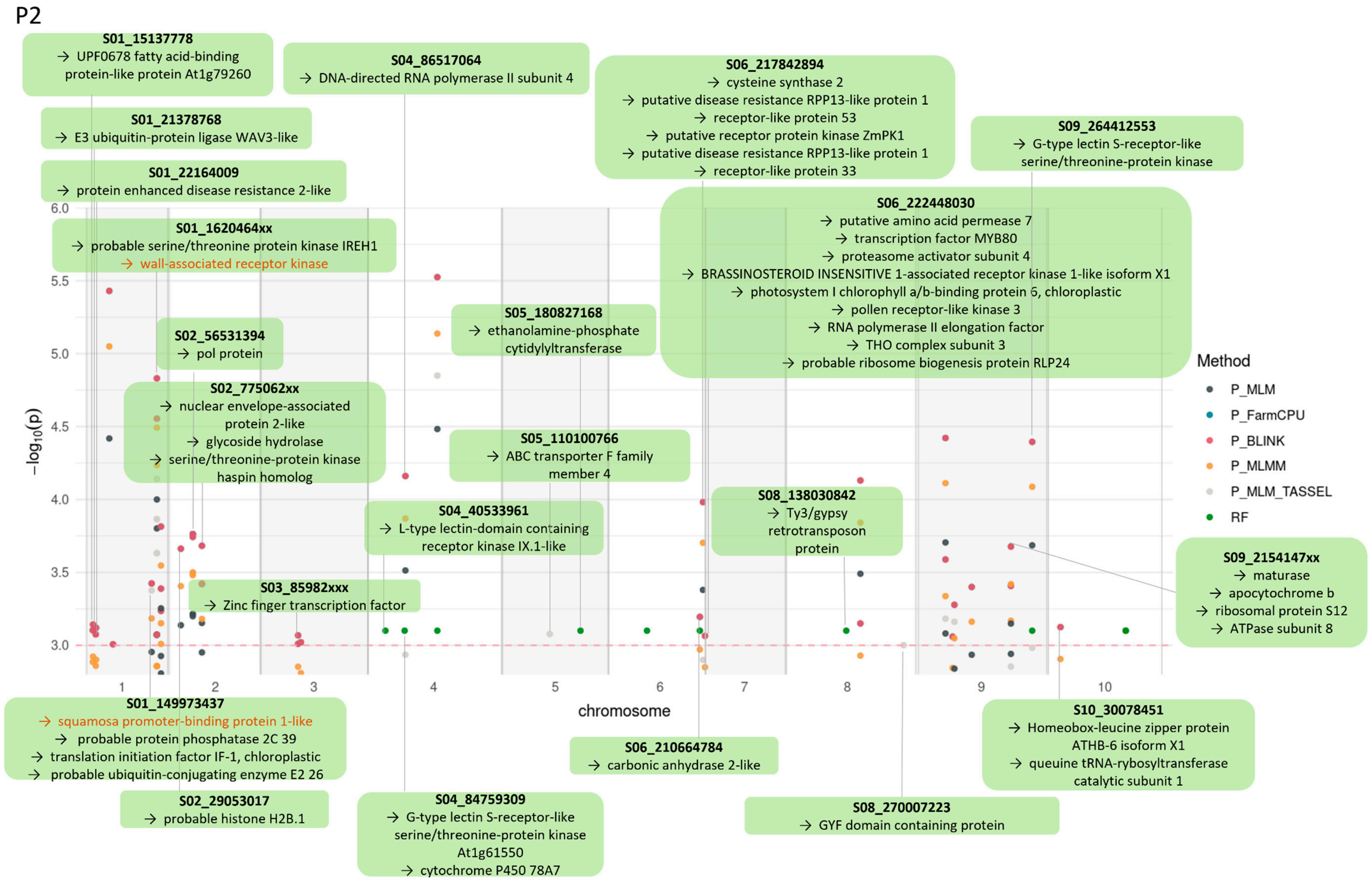

Figure S13.2: Manhattan plot of phase 2 with all the GWAS models with SNPs with p<0.001 (above dashed pink line) and top 10 important SNPs from Random Forest with artificial p value (-$\log_{10}$p=3.1) *for illustration. FarmCPU and BLINK p-values are denoted as BLINK values since they overlay. SNPs with annotated genes in the ±50 kbp region are marked and its BLAST descriptions from Supplement_11 are noted (→). Orange colour in the gene name indicates that the gene has SNPs on its coding region*
