# Supplementary material for: Association-Based Analysis of Verticillium Wilt Resistance in a Bi-Parental Hop (Humulus lupulus L.) Population for Marker Development in Breeding
Source: Plants (Basel). 2026 May 29;15(11):1667. doi: 10.3390/plants15111667 (PMC13259542; doi:10.3390/plants15111667)
Supplement: Supplementary file 1 [file plants-15-01667-s001.zip › Supplementary_file_S14.pdf]

Supplementary file S14: List of markers associated with Verticillium wilt resistance QTLs and their positions in the Apollo phased genome.

Microsatellite	Accession	Phase 1 (position on chromosome 6)	Phase 2 (position on chromosome 6)
HlAGA6	AY588400	40,510,713-40,511,131	42,085,781-42,086,246
HlAGA8	AY588402	43,263,609-43,264,038	44,525,537-44,525,960
EMHL052	AJ586798	51,412,977-51,413,564	51,869,486-51,870,092

Table S14.1: List of markers associated with Verticillium wilt resistance QTLs and their positions in the Apollo phased genome.
