# Supplementary material for: Association-Based Analysis of Verticillium Wilt Resistance in a Bi-Parental Hop (Humulus lupulus L.) Population for Marker Development in Breeding
Source: Plants (Basel). 2026 May 29;15(11):1667. doi: 10.3390/plants15111667 (PMC13259542; doi:10.3390/plants15111667)
Supplement: Supplementary file 1 [file plants-15-01667-s001.zip › Supplementary_file_S3.pdf]

Supplementary file S3: Filtering of VCF files and SNP distribution data.

	**Number of SNPs**	
**Filter**	Phase 1	Phase 2
none	57,074,317	38,417,611
1 (missing data per site < 35.5%)	1,129,345	882,433
2 (MAF=0.05; min het=0.2, max het=0.7)	27,277	36,127

Table S1: Number of SNPs per phase in the filtering steps.

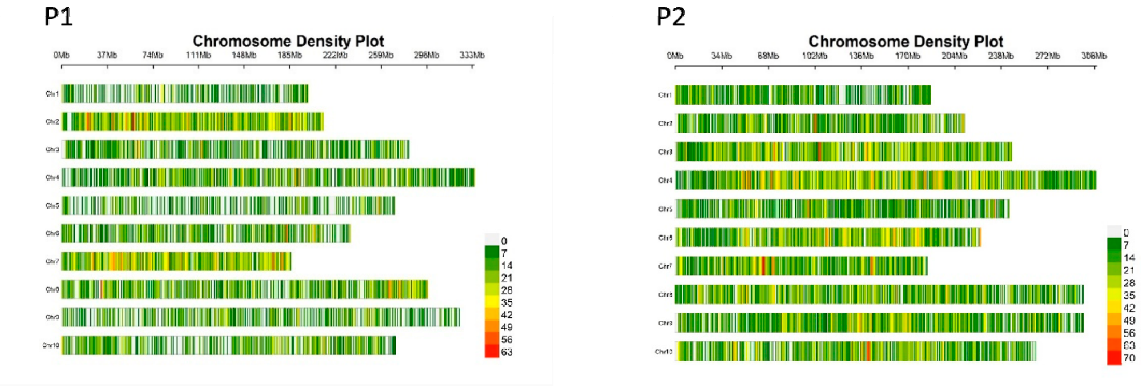

Figure S1: SNP distribution across chromosomes after filtering – for the .vcf files used in downstream analysis, designated by colours: green = low density, yellow = moderate, red = high density, calculated by the number of SNPs per 1 Mbp.

	Phase 1			Phase 2		
**CHR**	**Average SNP distance**	**Nr. of SNP**	**Average DP**	**Average SNP distance**	**Nr. of SNP**	**Average DP**
1	168,446	1,184	503	102,531	1,819	502
2	51,778	4,085	590	79,901	2,619	502
3	111,169	2,503	525	55,602	4,400	503
4	97,105	3,440	543	54,224	5,649	500
5	163,210	1,647	514	86,896	2,785	505
6	104,024	2,243	538	58,268	3,806	509
7	49,459	3,753	598	62,486	2,931	511
8	85,375	3,476	556	70,327	4,240	498
9	134,532	2,391	528	70,649	4,188	508
10	105,630	2,555	500	70,797	3,690	474
**All**	**95,289**	**27,277**	**548**	**67,786**	**36,127**	**501**

Table S2: Average filtered SNP distance and numbers per chromosome of each phase with average read depth at SNP genomic positions (DP).
