# Supplementary material for: Association-Based Analysis of Verticillium Wilt Resistance in a Bi-Parental Hop (Humulus lupulus L.) Population for Marker Development in Breeding
Source: Plants (Basel). 2026 May 29;15(11):1667. doi: 10.3390/plants15111667 (PMC13259542; doi:10.3390/plants15111667)
Supplement: Supplementary file 1 [file plants-15-01667-s001.zip › Supplementary_file_S4.pdf]

Supplementary file S4: Two figures of PCA analysis of Apollo genome phase 2 filtered variant file.

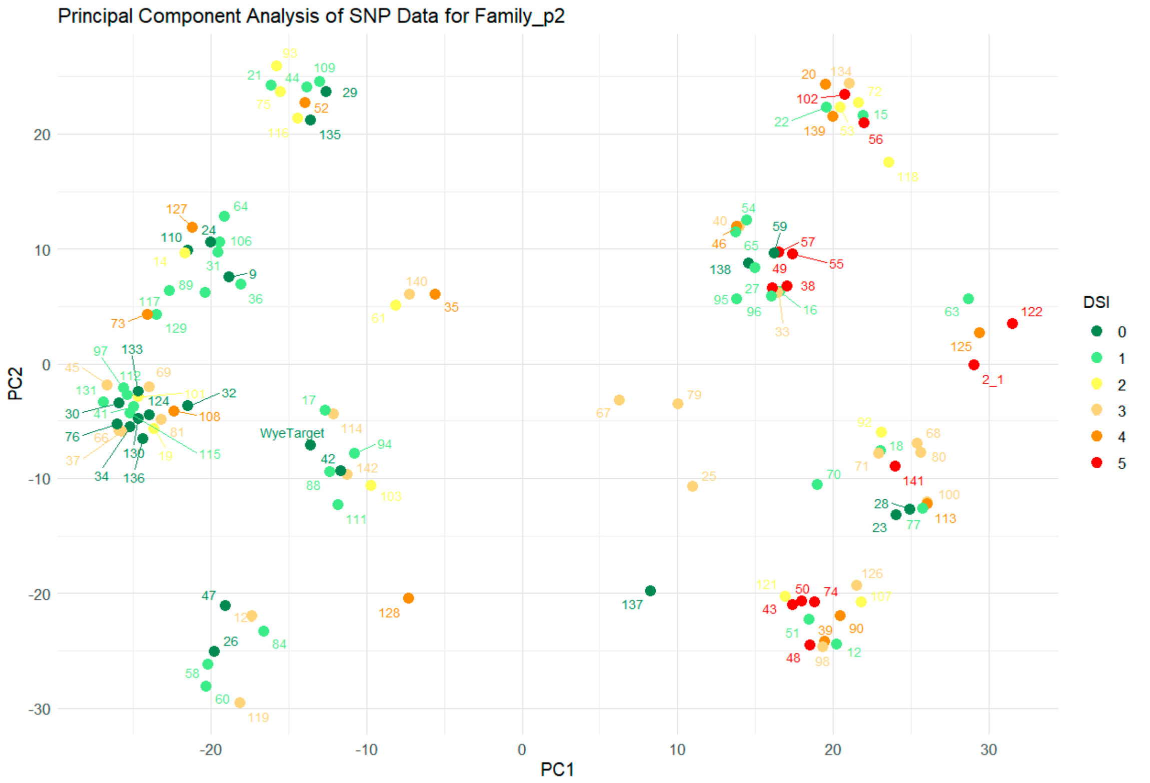

Figure S4.1: *PCA plot of 120 genotypes from family WT X BL2/1 with the parents on phase 2. Colour indicate DSI score.*

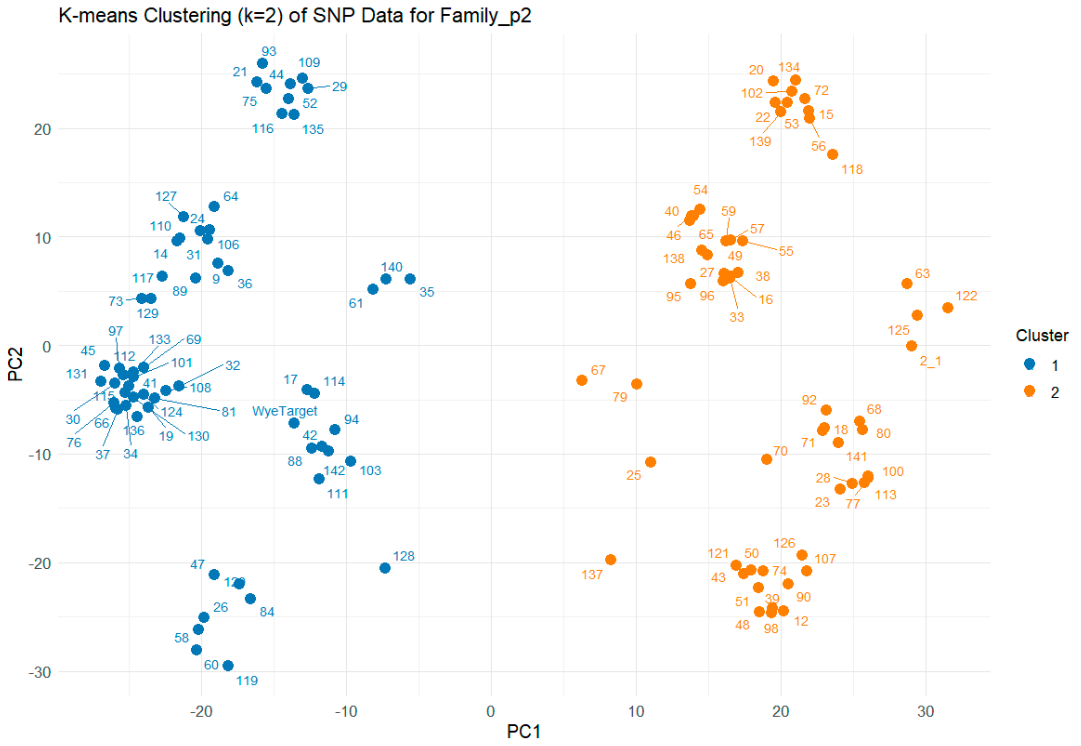

Figure S4.2: *PCA plot of 120 genotypes from family WT X BL2/1 with the parents on phase 2. Colour indicate k-means clustering (k=2). Mother WyeTarget is included in cluster 1 (blue) and father BL 2/1 in cluster 2 (orange).*
